# Cardioprotective Effects of Hydrogen Sulfide and Its Potential Therapeutic Implications in the Amelioration of Duchenne Muscular Dystrophy Cardiomyopathy

**DOI:** 10.3390/cells13020158

**Published:** 2024-01-15

**Authors:** Agnieszka Łoboda, Józef Dulak

**Affiliations:** Department of Medical Biotechnology, Faculty of Biochemistry, Biophysics and Biotechnology, Jagiellonian University in Krakow, Gronostajowa 7 Street, 30-387 Kraków, Poland; jozef.dulak@uj.edu.pl

**Keywords:** Duchenne muscular dystrophy, DMD, cardiomyopathy, iPSC-CM, induced pluripotent stem cell-derived cardiomyocytes, hydrogen sulfide, H_2_S

## Abstract

Hydrogen sulfide (H_2_S) belongs to the family of gasotransmitters and can modulate a myriad of biological signaling pathways. Among others, its cardioprotective effects, through antioxidant, anti-inflammatory, anti-fibrotic, and proangiogenic activities, are well-documented in experimental studies. Cardiorespiratory failure, predominantly cardiomyopathy, is a life-threatening complication that is the number one cause of death in patients with Duchenne muscular dystrophy (DMD). Although recent data suggest the role of H_2_S in ameliorating muscle wasting in murine and *Caenorhabditis elegans* models of DMD, possible cardioprotective effects have not yet been addressed. In this review, we summarize the current understanding of the role of H_2_S in animal models of cardiac dysfunctions and cardiac cells. We highlight that DMD may be amenable to H_2_S supplementation, and we suggest H_2_S as a possible factor regulating DMD-associated cardiomyopathy.

## 1. Duchenne Muscular Dystrophy: General Overview

Duchenne muscular dystrophy (DMD) is a neuromuscular condition caused by mutations in the *DMD* gene, resulting in a lack of dystrophin, a key component of the dystrophin-associated protein complex (DAPC) localized in the cortical cytoskeleton of skeletal myofibers and cardiomyocytes. DAPC, also known as the dystrophin–glycoprotein complex (DGC), not only links the actin cytoskeleton and the extracellular matrix [1,2], but also plays nonmechanical signaling roles; among others, it regulates neuronal nitric oxide synthase (nNOS) activity and calcium (Ca^2+^) homeostasis [3].

The first clinical presentation of DMD was described in the 1850s–1860s; however, it took more than one hundred years to identify fragments of the *DMD* gene cDNA (for references, see [4]). Currently, it is well known that the disease is caused by more than 7000 described mutations in the 79-exon-long *DMD* gene. The most common types of mutation are deletions of one or more exons (about 60–70% of all DMD cases), while point mutations constitute about 26% of cases and exonic duplication accounts for about 10–15%. Other modifications may also include missense mutations, splice mutations, and subexonic insertions or deletions. Despite differences in the type of mutations, all result in the absence of full-length dystrophin [1,5,6].

**Figure 1:**
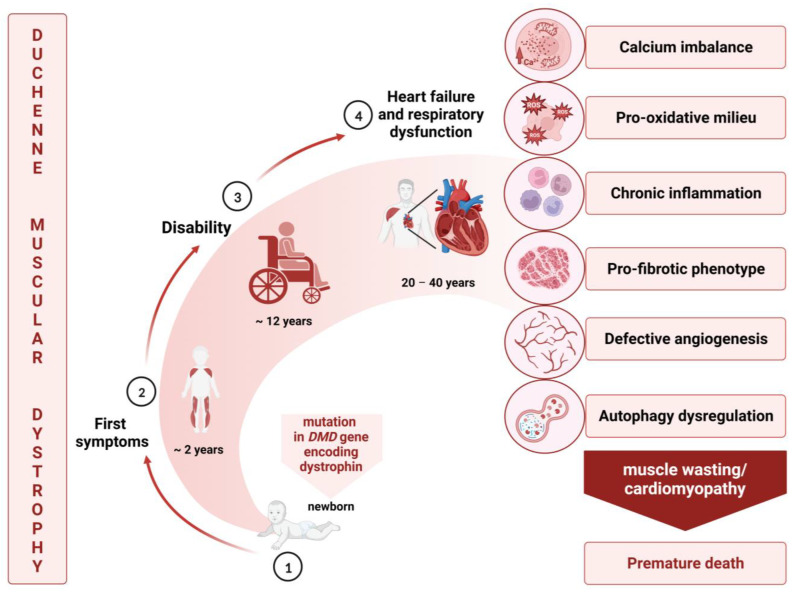
The course and main features of Duchenne muscular dystrophy. DMD is caused by mutations in the *DMD* gene ①, but symptoms are usually not evident until 2–3 years of age ②. The first signs, identified mostly by parents, typically include general motor delays and gait problems. Then, muscle weakness progresses and a wheelchair is required for almost all children, typically by about age 12 ③. Ultimately, disease progression leads to respiratory failure, cardiomyopathy, and premature death between the second and fourth decades of life ④. All these clinical features are the result of dystrophin deficiency and related complications, including calcium imbalance, increased oxidative stress, inflammation, and fibrosis, as well as dysregulated autophagy and angiogenesis.

DMD affects around 1:5000 male newborns, and in affected boys, dystrophin deficiency manifests with versatile and profound consequences [7]. Due to the instability of the sarcolemma and the related death of myocytes, patients with DMD suffer from progressive muscular weakness that results in loss of ambulation as teenagers, and further respiratory failure and cardiomyopathy that leads to premature death, mostly in the third decade of life [8] (Figure 1).

Crucial processes that contribute to disease progression include increased inflammation, oxidative stress, and fibrosis [9]; however, additional mechanisms, such as the dysregulation of angiogenesis or impaired autophagy, can also have an impact on this devastating condition. All these pathological events alter the myogenesis and regeneration process in dystrophic muscles, leading to a later loss of muscle tissues [10]. Unfortunately, there is no effective cure for DMD. Pharmacological treatment is mainly based on steroids, which counteract the processes that contribute to disease progression, but simultaneously exert many adverse effects. Their beneficial influence is attributed to a reduction in muscle inflammation, the prolongation of the independent walking period, and the prevention of scoliosis. Finally, their usage is correlated with postponed respiratory and cardiac changes [3]. To prevent or delay the deterioration of heart functions, several classes of drugs, such as angiotensin-converting enzyme inhibitors (ACEis), aldosterone receptor blockers (ARBs), β-adrenergic receptor (β-AR) blockers, and mineralocorticoid receptor antagonists (MRAs), are recommended. Their characteristics are described below, in Section 2.

Genetic approaches aimed at restoring the expression of functional dystrophin seem to be very attractive. However, the reconstitution of the full-length dystrophin cDNA, because of its size (>11 kb) and the limited capacity of the adeno-associated virus vectors (AAV) (around 5 kb), used most frequently as delivery tools, is technically challenging or even unfeasible. To avoid this obstacle, other strategies are proposed, including (i) the AAV-mediated delivery of truncated forms of dystrophin, called mini/micro-dystrophin, that contain only the domains required for their functions; (ii) the application of antisense oligonucleotide (AON) sequences to restore the open reading frame of dystrophin mRNA (exon-skipping strategy); (iii) enhancing the ribosomal readthrough of premature stop codons on dystrophin mRNA through the action of readthrough compounds, thus allowing production of the full-length protein; and (iv) the clustered, regularly interspaced short palindromic repeats (CRISPR)/CRISPR-associated protein 9 (Cas9) gene-editing system for functional restoration of the *DMD* gene (all these strategies are reviewed in [3]). Unfortunately, some of these approaches (exon skipping) can be applied to only a limited number of patients, as they could be/are mutation-specific. Currently, several such drugs have been approved by the Food and Drug Administration (FDA). Eteplirsen allows the skipping of exon 51; golodirsen and viltolarsen bypass exon 53, and casimersen is predicted to be used in DMD patients who have a confirmed exon 45 amenable mutation. Of note, golodirsen and casimersen are approved under accelerated review [11]. Similarly, recently (22 June 2023), Sarepta Therapeutics, Inc. announced that the FDA granted accelerated conditional approval for Elevidys, a gene therapy leading to the production of micro-dystrophin.

Although there is constant progress in the field of gene therapy as well as cardiac-directed treatments, there is still an urgent need to search for new approaches that could improve the life conditions of patients with DMD. As not only skeletal muscle weakness but mostly respiratory and cardiac muscle failure lead to premature death, the ideal therapeutic factor should target all diseased tissues.

## 2. Cardiovascular Complications in Duchenne Muscular Dystrophy

Despite recent advances in medical and interventional therapies, heart problems remain the leading cause of mortality and morbidity in DMD patients. Changes in heart functioning are evident very early during disease progression, and it is suggested to start cardiac-focused treatment even before signs of heart dysfunction are confirmed [12]. The first symptoms of cardiac failure may be present even in patients younger than 6 years of age and manifest themselves mainly as dilated cardiomyopathy (DCM), which can then turn into end-stage heart failure (HF) along with associated supraventricular and ventricular arrhythmias [13].

The lack of dystrophin in cardiomyocytes causes problems similar to those in skeletal muscle cells with increased cardiomyocyte structural vulnerability, membrane instability, and intracellular Ca^2+^ overload. The dysregulation of calcium homeostasis is critical for other events and results in improper contraction, leading, with the involvement of additional mechanisms, to cardiomyocyte hypertrophy. Calcium imbalance triggers a cascade of detrimental events, including a high production of reactive oxygen species (ROS), membrane depolarization, the persistent opening of the mitochondrial permeability transition pore (MPTP), and mitochondrial-mediated cell death related to the reduced ATP production and bioenergetic collapse. The infiltration of inflammatory cells and the fibrotic cascade also contribute to DCM development. The progressive impairment in the electrical conduction system leads to arrhythmias associated with DCM [14,15].

Although it is generally accepted that patients with DMD may benefit from heart-directed therapies, there is currently no complete agreement on how to treat DMD cardiomyopathy. However, there is a consensus that early diagnosis and routine follow-up visits with a cardiologist should be standard procedures for DMD individuals [12].

Several drugs were shown to have beneficial effects in DMD boys (Table 1). Angiotensin-converting enzyme inhibitors (ACEis) inhibit angiotensin II binding to the angiotensin 1 receptor (AT_1_) and limit its stimulatory effect on oxidative stress, heart fibrosis, and cardiomyocyte death [16]. Initially, it was suggested that ACEis should be the first-line therapy once left ventricular (LV) dysfunction has developed; however, nowadays, it is recommended that treatment with such drugs, like perindopril, enalapril, and captopril, should start even before the onset of LV dysfunction [17]. In a 10-year follow-up study, presymptomatic treatment with perindopril for 3 years was shown to result in a significantly higher survival rate [18]. Also, as substitute compounds, angiotensin receptor blockers (ARBs) exert cardioprotective effects in DMD individuals and can be used especially in those who are intolerant to ACEis [19]. For example, losartan, belonging to this class, was shown to have beneficial hemodynamic effects in patients with HF [20]. Taking all this into consideration, the National Heart, Lung, and Blood Institute (NHLBI), in collaboration with the Parent Project Muscular Dystrophy, recommended the use of ACEis or ARBs even in asymptomatic DMD boys with normal LV systolic function by the age of 10 years [21].

Other heart-orientated drugs with possible applications in DMD are mineralocorticoid receptor antagonists (MRAs) (or aldosterone antagonists). Spironolactone and eplerenone exert cardioprotection in DMD patients and attenuate the decline in cardiac function [22,23,24]. The timing of the use of these drugs is not strictly defined; however, early MRA treatment should be considered to maximize the chance of cardiac improvement.

Beta-adrenergic receptor (β-AR) blockers such as bisoprolol, metoprolol, and carvedilol [25,26,27] are considered second-line therapy in patients with tachycardia and/or no effect of ACEis. Importantly, the combination of ACEis with β-blockers can have additional plausible effects, outweighing a single treatment, with an improvement in LV systolic function and overall outcomes (reviewed in [28]). Moreover, a recent analysis by Lechner et al. [29] showed that the concomitant use of ACEis, β-blockers, and aldosterone antagonists was significantly associated with a lower LV ejection fraction in patients with DMD.

To comprehensively understand the molecular processes underlying heart dysfunctions in patients, as well as to test preventative and treatment regimens, the use of animal models is essential. Both non-mammalian (*Caenorhabditis elegans*, *Drosophila melanogaster*, and *Danio rerio*) and mammalian animal models of DMD can be used to investigate skeletal and cardiac pathologies (reviewed in [30]). Although murine models are frequently used in DMD studies, modeling heart dysfunction in dystrophic mice is challenging. The most frequently used *mdx* mice with a spontaneous mutation in exon 53 of the *Dmd* gene do not develop the cardiac phenotype found in human DMD individuals, with fibrosis and dysfunction developing late, if at all. Quinlan et al. [31] found that young animals have normal cardiac function, and with age, hypertrophic cardiomyopathy develops, but even in very old (17-month-old) mice, only eight percent of the heart muscle is fibrotic.

The differences between mouse and human cardiac complications may result from the unique adaptiveness of the *mdx* heart to dystrophic conditions that delay cardiac pathology. Particularly, an increase in the activity of heart mitochondria through the high Ca^2+^ uptake rate and its transport, possibly compensating for a defective sarcoplasmic reticulum, was found [32,33,34]. Moreover, the overactivation of respiration and high intensity of oxidative phosphorylation [35] may mask the development of destructive processes and fibrosis in the early stages of the development of cardiomyopathy [36,37], differentiating *mdx* mice from human patients.

Another constraint on the mouse model is the sex disparity between male and female *mdx* mice. In contrast to the slower disease progression observed in female human carriers [38], in *mdx* mice, more prominent cardiac abnormalities have been found in females than in males [39,40]. However, because DMD is almost uniquely present in human boys and young men (female patients with a lack of two *DMD* alleles are sporadic; the female carriers represent another disease category), all data generated through investigations using female mice should be addressed with proper caution with regard to their relevance to the human situation.

As a result of these difficulties, some other mouse models were created, displaying a noticeably more pronounced cardiac phenotype. In D2-*mdx* mice, myocardial inflammation and calcification at 7 weeks of age are detected, while a decreased LV ejection fraction and fractional shortening at 6 months of age are evident. However, it was demonstrated that in wild-type (DBA/2J) control animals, cardiac fibrosis also develops, especially in older animals, which can limit the usefulness of this strain to model cardiac pathology. More severe cardiac abnormalities have been detected in the *mdx-Cmah^−/−^* model (mice carrying a human-like mutation in the mouse *Cmah* gene as well as a mutation in *Dmd*) and *mdx/Utrn^−/−^* mice (devoid of dystrophin and utrophin production), making them more appropriate to study the role of H_2_S in cardiac events. Moreover, Mourkioti et al. [37] hypothesized that the differences in *mdx* and DMD cardiac dysfunction are caused by the variability in telomere length between mice and humans. Indeed, *mdx* mice lacking the RNA component of telomerase (*mdx*/mTR) develop severe functional cardiac deficits with fibrosis and dilated cardiomyopathy, leading to early death from HF that is similar to DMD patients (reviewed in [15,30,41]). 

Larger animals could be of special interest as they better resemble human disease; however, they may also display some unique characteristics. Dystrophic rabbits, cats, dogs, and even pigs were used to recapitulate human pathology. Among them, the canine DMD model (golden retriever muscular dystrophy (GRMD) dogs with a spontaneous splice site mutation in the *DMD* gene) exhibits not only histological abnormalities, but also an overall clinical course that is strikingly comparable to those of DMD patients. However, its use is limited due to practical issues such as high costs and the number of animals available [42]. Finally, porcine models of DMD have been generated. The deletion of exon 52 of the *DMD* gene, a mutation frequently present in humans, through a targeted replacement of exon 52 with a neomycin resistance cassette (*neo*^®^), led to the generation of DMD^∆52^ pigs with typical signs of DMD, including mobility impairment and severe myopathy [43]. This model was also used to assess the effectiveness of *DMD* gene editing. Using CRISPR/Cas9 technology, Moretti et al. demonstrated that the restoration of a shortened dystrophin (DMDΔ51–52) in porcine muscles resulted in improved functions, reduced arrhythmogenic vulnerability, and prolonged survival [44].

Although various animal DMD models are available, as discussed above, they do not fully reproduce human pathology. The use of human induced pluripotent stem cells (hiPSCs) differentiated into the specified cells may be helpful in the evaluation of the mechanisms of DMD. It is well-established that iPSC technology offers a unique opportunity to generate human cells and investigate pathological conditions in a personalized way. Due to their properties, hiPSCs can be used to obtain patient-specific stem cells, and by differentiating into cells affected by the patient’s disease, for example, cardiomyocytes, they can be used to study disease mechanisms and drug/compound testing. Therefore, hiPSC-derived cardiomyocytes represent an excellent tool for modeling cardiac dysfunctions, including DMD-associated cardiomyopathy. This is especially important in knowing that the collection of a heart biopsy is not a routine diagnostic procedure, and human cardiomyocytes are post-mitotic cells; hence, their long-term culture, even if biopsy is available, is not possible. 

hiPSC-derived cardiomyocytes (hiPSC-CMs) may replace or constitute animal models of cardiac impairment in DMD. This technology was used in our [45,46,47,48] and other (reviewed in [49]) studies. To model DMD, we have created isogenic control hiPSCs, and through the CRISPR/Cas9-mediated deletion of *DMD* exon 50, we obtained dystrophin-deficient lines. We also corrected the mutation in the patient-derived hiPSCs [48]. Dystrophic hiPSC-CMs showed impaired iron homeostasis and decreased levels of mitoNEET, a mitochondrial protein responsible for the maintenance of energy homeostasis and the regulation of labile iron levels in mitochondria, which led to increased oxidative stress, a common DMD pathology. We also demonstrated that deferoxamine (an iron chelator) or pioglitazone (the compound that stabilizes mitoNEET) decreased the level of ROS in DMD hiPSC-CMs. In dystrophic hiPSC-CMs, the electrophysiological abnormalities were evident, and they were corrected by the iron chelator and mitoNEET stabilizer [48]. Similarly, Kamdar et al. showed cardiac arrhythmias in dystrophic hiPSC-derived cardiomyocytes compared to isogenic controls [50]. Additionally, they found that β-blockers reduced the incidence of arrhythmogenesis. Moreover, Eisen et al. [51] revealed a number of electrophysiological alterations, including abnormal action potential parameters, an increased beat rate variability, and arrhythmogenic firing patterns, not only in hiPSC-CMs generated from a DMD patient but also from a symptomatic carrier female.

It should be noted that the heart consists not only of cardiomyocytes, and the dysfunction of other dystrophin-deficient cells, including endothelial cells, vascular smooth muscle cells, and fibroblasts [52,53,54,55], may add to heart problems in DMD patients. hiPSC technology can be helpful in obtaining all of these cell types. Moreover, 3D cultures, spheroids, and organoids composed of various cells might better mimic the dystrophic microenvironment and connections between cells in the DMD heart [56].

Altogether, it implies that both animal models and hiPSC technology may be applied to study the cardiovascular complications in DMD. They could also be used to assess the potential of possible therapeutic agents, one of which may be hydrogen sulfide. 

## 3. Hydrogen Sulfide—Does the Method of Delivery Matter?

H_2_S is a colorless, flammable, water-soluble gas with a strong rotten egg smell. Despite being regarded mostly as a lethal toxic pollutant in the past, it is now well-recognized as an endogenously produced, labile, diffusible mediator with robust pleiotropic effects. The possibility of modulating a myriad of biological signaling pathways suggests its indispensable role in both health and disease. The results of many studies indicate a correlation between the low concentration of endogenous H_2_S levels and pathophysiological conditions. Not only neurological disorders, such as Alzheimer’s disease, but also diabetes, obesity, asthma, some cardiovascular complications, and cancers may be influenced by the dysregulated level of this gasotransmitter [57].

**Figure 2:**
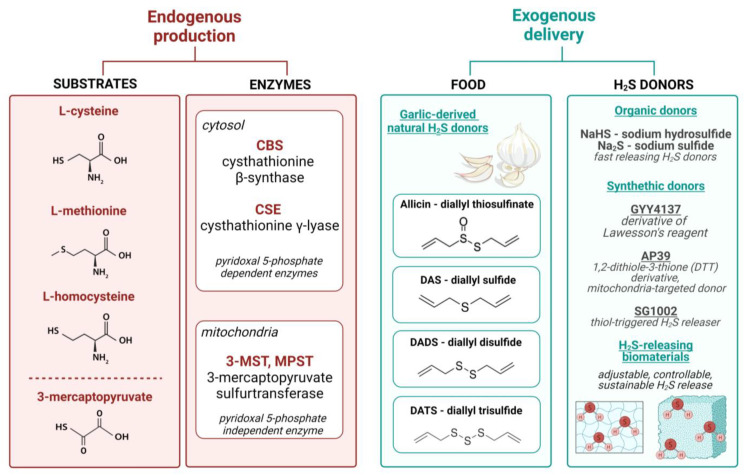
Simplified scheme of endogenous production of H_2_S and examples of natural and synthetic donors. H_2_S is produced enzymatically, mainly from L-cysteine, L-methionine, and L-homocysteine, via the activity of cystathionine-β-synthase (CBS) and cystathionine-γ-lyase (CSE), and from 3-mercaptopyruvate via 3-mercaptopyruvate sulfurtransferase (3-MST, MPST). For exogenous delivery, compounds present in garlic, like allicin and its derivatives, as well as various forms of synthetic donors, can be used. Recently, H_2_S-releasing biomaterials have been tested for adjustable and controllable gas delivery.

In mammals, H_2_S is produced enzymatically via different pathways. L-cysteine, L-methionine, and L-homocysteine serve as the main substrates for cystathionine-β-synthase (CBS), mostly in the nervous system, brain, and liver, as well as cystathionine-γ-lyase (CSE), expressed predominantly in the cardiovascular system. Another pathway relies on the activity of 3-mercaptopyruvate sulfurtransferase (3-MST, MPST), primarily localized in mitochondria, which catalyzes the production of H_2_S from 3-mercaptopyruvate (3-MP) (Figure 2). 3-MP can be generated not only from L-cysteine by cysteine aminotransferase (CAT), but also from D-cysteine through D-amino acid oxidase (DAO) activity [58].

Importantly, due to the difficulty in achieving precisely regulated concentrations and the potentially harmful impact of H_2_S excess, its direct delivery in the gaseous form is not an ideal method of its upregulation. Therefore, to increase the level of this mediator, a great number of donors with different chemistry affecting release profiles, water solubility, in vivo bioavailability, and the renal clearance rate have been tested.

Some naturally existing compounds (Figure 2), allicin (diallyl thiosulfinate) and its derivatives, such as diallyl sulfide (DAS), diallyl disulfide (DADS), and diallyl trisulfide (DATS), are thought to be responsible for a range of the health benefits of garlic. These organosulfur compounds differ in the chemistry of H_2_S release, with DATS reacting rapidly with glutathione (GSH) to release H_2_S through the thiol–disulfide exchange, while DADS produces a trace quantity of H_2_S in a slow reaction with GSH via the α-carbon nucleophilic substitution pathway [59].

Differences in H_2_S release are also found between various H_2_S donors. Sodium hydrosulfide (NaHS) and sodium sulfide (Na_2_S), conventional inorganic donors, were first used, but recently, synthetic compounds, with the controllable release of the gas or specific, organelle-targeted donors, are of much interest for the application in in vitro and in vivo studies (Figure 2). 

Although widely used in vitro, NaHS and Na_2_S have limited applications due to the fast and uncontrolled release of H_2_S. In line with that, various studies have demonstrated the benefits of short-term inorganic donor delivery, including its cardioprotective effects in murine [60] and porcine [61] models of myocardial infarction (MI). Interestingly, some evidence indicates the effectiveness of long-term Na_2_S therapy. Ischemia-induced heart failure, induced by subjecting C57BL6/J mice to 60 min of left coronary artery occlusion followed by reperfusion for up to 4 weeks, was attenuated by Na_2_S administration once at the time of reperfusion and then daily for the first 7 days of reperfusion [62]. Despite such results, attempts have been made to synthesize compounds boosting H_2_S concentration slowly and in a more controlled manner. For example, water-soluble GYY4137 (morpholin-4-ium 4 methoxyphenyl(morpholino) phosphinodithioate), a pH-sensitive donor, releases this potent gasotransmitter significantly slower (10 min) compared to NaHS (10 s). There are also donors with other modalities, such as enzyme-activated compounds, ROS-activated molecules, and photo-induced or thiol-triggered donors (reviewed in [63]). The last group can be represented by the H_2_S prodrug, sodium polysulthionate (SG-1002), which provides a sustained increase in H_2_S levels, as demonstrated in the phase I clinical trial in patients with congestive heart failure [64]. This compound can be taken orally and is a promising candidate for further clinical studies. For organelle-specific H_2_S upregulation, AP39, the mitochondria-targeted and slow-releasing donor, might be considered [65]. Targeting mitochondria significantly improves its effectiveness, even when used at nanomolar concentrations (while other donors are mostly applied at much higher, micromolar doses). The delivery of H_2_S to mitochondria can also increase its cardioprotective properties, which have been demonstrated, for example, in a rat model of ischemia/reperfusion (I/R) injury through hampering mitochondrial ROS production [66]. 

Still, the area of chemical modifications of H_2_S donors is not fully explored. Attempts are made to reduce their toxicity and improve their renal clearance and increase their water solubility and, therefore, their bioavailability. Recent discoveries focus on the preparation of H_2_S-releasing biomaterials to provide adjustable and controllable, as well as sustainable, H_2_S release. In this approach, biological scaffolds are loaded with various H_2_S donors through chemical conjugation or physical incorporation. For example, metal–organic frameworks (MOFs), including Zn-based MOFs to store and deliver H_2_S, have been generated [67]. Additionally, localized and prolonged H_2_S delivery could be achieved with the use of hydrogels (especially those that are implantable), prepared from collagen, alginate, hyaluronic acid, and other materials. H_2_S donors could be encapsulated into hydrogel matrices or linked covalently into the hydrogel network. A detailed description of the chemical synthesis and properties of such scaffolds can be found in a recent review [68]. 

As the field of synthetic H_2_S donors advances rapidly, high-quality compounds characterized by well-defined releasing mechanisms, activated on demand and releasing gasotransmitters locally, may soon (hopefully) be offered for clinical applications.

## 4. Hydrogen Sulfide—A Cytoprotective Gas

**Figure 3:**
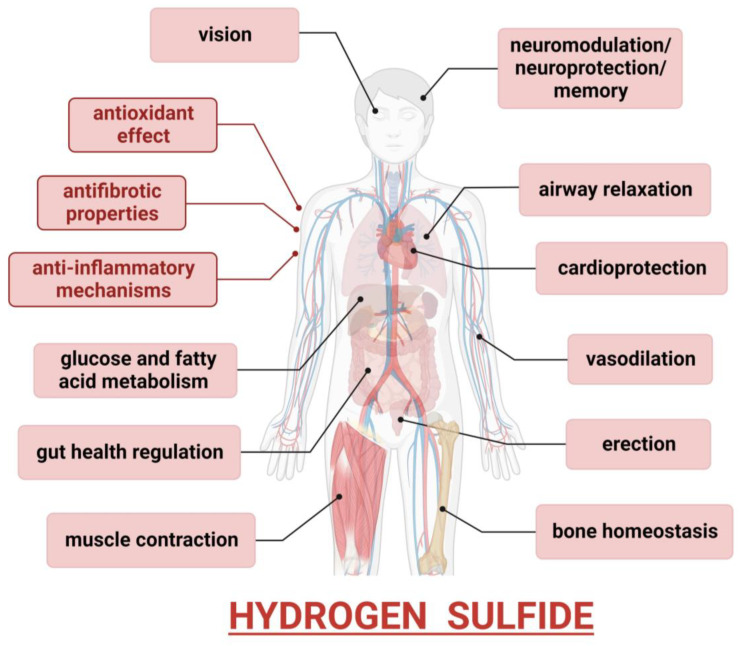
Physiological roles of H_2_S. H_2_S may regulate the functions of various organs in the human body. Antioxidant, anti-inflammatory, and anti-fibrotic properties could be exerted in many tissues. Moreover, it may play organ-specific effects in the brain, lungs, heart, liver, gastrointestinal tract, reproductive system, bones, and skeletal muscles.

Hydrogen sulfide is a critical regulator in many biological processes and exerts a plethora of effects on mammalian physiology, acting as a neurotransmitter; a regulator of vasodilation and vascular tone; and a modulator of inflammatory, antioxidant, pro-survival, and anti-apoptotic signaling. The neuromodulatory role of H_2_S, mostly through the regulation of the N-methyl-D-aspartate (NMDA) receptor responses, is well evidenced, and its potential neuroprotection in the development of neurodegenerative diseases is thoroughly described in a recent review paper [69]. The effects of this gasotransmitter on the proper functioning of other organs, including the lungs, liver, gastrointestinal tract, bones, and skeletal muscles, through a variety of mechanisms, are also well documented (Figure 3). Accordingly, it is also evident that the dysregulation of H_2_S levels may be implicated in the development and/or progression of neurodegenerative disorders, cardiovascular and pulmonary diseases, erectile dysfunctions and reproductive abnormalities, osteoporosis, and other bone-related pathological conditions, as well as myopathies. 

To study the molecular mechanisms of H_2_S-mediated cardioprotection, in vitro studies utilizing cardiomyocytes have been performed, including rat H9C2 cells [70,71,72,73], murine HL-1 cells [74,75], or human AC16 [76] (Table 2). Interestingly, scarce information has been published on the use of hiPSC-CMs [77].

Nevertheless, the analysis of published studies in which various exogenous donors, like NaHS [70,71,72,73,75,77], SG-1002 [74], and GYY4137 [75], were used (discussed broader below) may provide some insight into their possible beneficial effects in dystrophic cardiomyocytes, the heart, and muscles.

## 5. Molecular Mechanisms of Cardioprotective Effects of Hydrogen Sulfide

Undoubtedly, an important aspect of H_2_S’s functions is related to its activity in the cardiovascular system, mainly the regulation of vascular tone, the relaxation of blood vessels, and lowering blood pressure. Many studies have shown a downregulation of H_2_S signaling in cardiovascular diseases, like heart failure, ischemic myocardium, atherosclerosis, and hypertension, while supplementation with H_2_S donors was protective against these cardiac-related events. These results are discussed in the following sub-chapters.

### 5.1. H_2_S Regulates the Activity of Ion Channels

Numerous studies concentrate on the evaluation of molecular pathways in the cardioprotective effects of H_2_S. The first identified and primary H_2_S targets are ATP-sensitive potassium (K_ATP_) channels [85], which can serve as metabolic sensors and are highly sensitive to ATP and ADP concentrations. High intracellular concentrations of ATP inhibit channel activity. In a physiological state, a high intracellular concentration of ATP results in a closed and inactivated channel state, whereas under stressful conditions, the ATP concentration in myocardial cells decreases, causing the opening of K_ATP_ channels, and the outflow of K^+^ increases. In consequence, Ca^2+^ inflow is reduced and myocardial contraction is inhibited [86]. 

In pathological situations, including hypertension [87], ischemic heart disease [88], dilated cardiomyopathy [89], or cardiomyopathy in DMD [90], a disruption of the functions of the K_ATP_ channel has been evident. On the other hand, K_ATP_ activation may prevent uncontrolled calcium influx, stabilize membrane potential, and regulate cardiac contractility [91]. Accordingly, the activation of K_ATP_ by H_2_S protects the myocardium from the detrimental effects of I/R injury [92], increased blood vessel dilation, and decreased blood pressure [93]. 

K_ATP_ channels are built from pore-forming subunits, Kir6.x (Kir6.1 or Kir6.2), and a sulfonylurea receptor (SUR1, SUR2, SUR2A, SUR2B), having regulatory activity. H_2_S may affect the activity of the specific subunits in a cell type-dependent manner. For example, in colonic smooth muscle cells, the opening of SUR2B [94] was evident, while in vascular smooth muscle cells, H_2_S targeted the SUR1 subunit [95].

Depending on their sublocalization, K_ATP_ channels can be present in the sarcolemma, mitochondria, and nucleus, and are thus referred to as sarcolemmal (sarcK_ATP_), mitochondrial (mitoK_ATP_), and nuclear (nucK_ATP_), respectively. There are studies indicating that both sarcK_ATP_ and/or mitoK_ATP_ channels can be regulated by H_2_S. The beneficial, anti-apoptotic, and pro-survival effects of NaHS were blunted after the inhibition of mitochondrial K_ATP_ [96]. Consistent with this finding, Testai et al. [97] demonstrated that the other H_2_S donor (4-carboxy phenyl-isothiocyanate, 4CPI) had no favorable effects on the post-ischemic recovery of the rat myocardium after applying the mitoK_ATP_ channel blocker. Bian et al. [98], on the other hand, emphasized the relevance of sarcK_ATP_ channels in H_2_S effects. NaHS treatment enhanced cardiac myocyte viability and lowered the severity and duration of arrhythmias following I/R. By inhibiting both the sarcK_ATP_ and mitoK_ATP_ channels, it was evidenced that the latter did not mediate the beneficial effects of NaHS [98]. 

Of importance, K_ATP_ activators have been shown to exert beneficial effects on the development of DMD, including cardiac pathology. Uridine, the pyrimidine nucleoside and the precursor of UDP, the activator of K_ATP_ channels, improved the functioning of the mitochondrial K^+^ channels in *mdx* animals [99]. Similarly, nicorandil, an NO donor and a K_ATP_ channel opener, prevented stress-induced dystrophin-deficient cardiomyocyte death and maintained *mdx* cardiac functions after ischemia/reperfusion damage [100].

Not only K_ATP_ channels are involved in the cytoprotective effect of H_2_S. In addition, the activation of K_v_7 voltage-gated potassium channels (particularly K_v_7.4) [101] and the inhibition of the large-conductance calcium-activated potassium (BK) channels [102] may mediate the vasorelaxant activity of H_2_S. The regulation of the activity of ion channels has also been demonstrated in hiPSC-CMs [77]. Interestingly, a patch-clamp electrophysiological measurement showed the inhibitory effect of NaHS on action potential, outward rectifier potassium currents (slow I_Ks_ and rapid I_Kr_), and L-type Ca^2+^ currents (I_CaL_) in this model. Moreover, the inhibition of the potassium channel Kv1.5 in HL-1 cardiomyocytes by NaHS and GYY4137 donors was also evident [75]. Similarly, in rat cerebral arteries, the inhibition of I_CaL_ channels appears to be a crucial vasculoprotective mechanism of NaHS [103], while in rat coronary arteries, the activation of 4-aminopyridine (4-AP)-sensitive potassium channels has been involved in the vasorelaxant activity of the same H_2_S donor [104].

In *mdx* mice, a decrease in the expression of the mitochondrial BK channels was evident, while the restoration of their activity had a positive effect on the state of the skeletal muscles. Dubinin et al. [105,106] demonstrated that NS1619, the BK activator, normalized potassium transport, increased calcium retention capacity, decreased oxidative stress, and improved mitochondrial structure. The crosstalk (if any) between BK channel regulation by H_2_S in DMD cardiomyopathy is not known. Further studies should focus on a more detailed evaluation of the H_2_S effect on the activity of various ion channels and its implication for cardiac hemodynamics.

### 5.2. S-Sulfhydration Contributes to H_2_S-Triggered Cardioprotection

**Figure 4:**
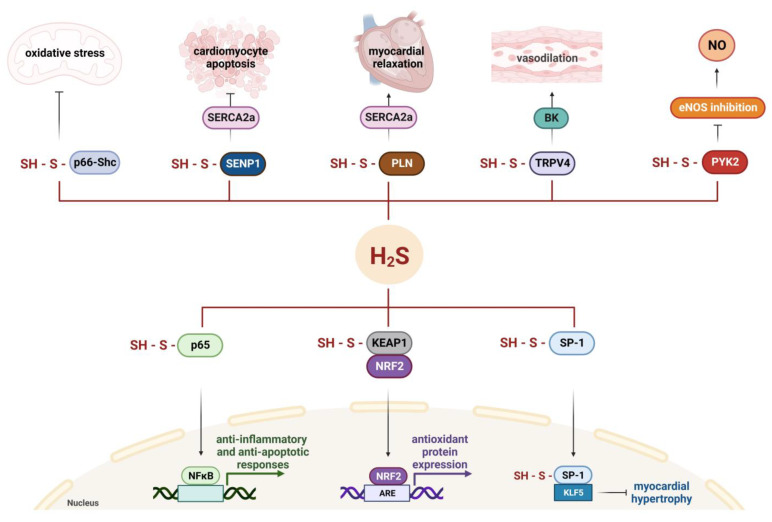
S-sulfhydration represents an important mechanism of H_2_S-mediated cardioprotection. H_2_S sulfhydrates p66Shc and KEAP1 to inhibit oxidative stress, NF-κB to halt inflammation and apoptosis, SP-1 to protect against myocardial hypertrophy, and transient receptor potential V4 (TRPV4) to trigger vasodilation. Regulation of Ca^2+^-ATPase (SERCA2a) may result from S-sulfhydration of sentrin-specific protease 1 (SENP1) or phospholamban (PLN). The conversion of an -SH group to an -SSH in the protein tyrosine kinase 2 (PYK2) decreases its inhibitory activity on endothelial nitric oxide synthase (eNOS) activity and NO production, which results in the broad cytoprotective effect of H_2_S. ARE—antioxidant response element; BK—large-conductance Ca^2+^-activated K^+^ channel; KLF5—Krüppel-like factor 5; NRF2—nuclear factor erythroid 2-related factor 2.

The pleiotropic nature of H_2_S and its cardioprotective activities, including antioxidant, anti-inflammatory, and anti-apoptotic effects, detected under various experimental conditions, can depend on the possibility of triggering oxidative modifications, including persulfidation, a post-translational modification of cysteine residues (RSHs) to persulfides (RSSHs), of specific macromolecules, such as proteins, redox-sensitive factors, and signaling complexes [107]. Recent studies identified detailed cysteine sites subjected to S-sulfhydration in several proteins regulating cardiac relaxation or hypertrophy, vasodilation, oxidative stress, inflammation, and apoptosis (Figure 4).

As mentioned above, the regulation of ion channels represents the main mechanism contributing to H_2_S-mediated cardioprotection. Importantly, this effect may result from S-sulfhydration. The Kir6.1 subunit of K_ATP_ was shown to be S-sulfhydrated at Cys43 after NaHS treatment in HEK293 cells, leading to increased K_ATP_ channel activation and vasodilation [108]. Moreover, in human aortic endothelial cells, NaHS also sulfhydrated IK_ca_ channels, which may contribute to H_2_S-dependent hyperpolarization [108]. In addition to the post-translational modification of the pore-forming Kir6.1 subunit, another subunit of the K_ATP_ channel complex, the sulfonylurea receptor, SUR2B, with regulatory activity, was also shown to be sulfhydrated [109]. Interestingly, while the SUR2B sulfhydration of Cys24 and Cys 1455 was induced by NaHS treatment, this H_2_S donor did not modify any cysteine residue in the Kir6.1 subunit in mouse colonic smooth muscle cells. Nevertheless, when the SUR2B subunit was sulfhydrated, the tyrosine nitration of the Kir6.1 subunit was abolished, having beneficial effects on colonic inflammation [109]. The other example could be the transient receptor potential V4 (TRPV4) channel, belonging to the bigger family of proangiogenic Ca^2+^-permeable TRP channels. Naik et al. [110] demonstrated an increased S-sulfhydration of TRPV4 following Na_2_S treatment in aortic endothelial cells. This mechanism is responsible for TRPV4 activation, calcium influx, and the opening of BK channels, resulting in endothelial hyperpolarization and subsequent vasodilation (Figure 4). All these data indicate that the regulation of ion channel activity by H_2_S greatly contributes to the vasorelaxant effects of this gaseous transmitter; however, S-sulfhydration may be cell-, donor-, and environment (physiological or pathological)-dependent.

The other mechanism of the cardioprotective effects of H_2_S relies on the regulation of the activity of soluble guanyl cyclase (sGC) and an increase in the level of cGMP. H_2_S is capable of inhibiting cGMP phosphodiesterase (PDE5) activity, which increases the half-life of cGMP [111]. These effects are similar to those of NO activity, and crosstalk between these two gasotransmitters is likely to occur. Of note, Chatzianastasiou et al. [112] demonstrated that, although different donors, such as Na_2_S and AP39, exerted cardioprotective activities in the I/R injury model and decreased infarct size to a comparable degree, AP39 worked independently of the NO/cGMP pathway. In contrast, the activity of Na_2_S involved NO signaling. Similar cardioprotective effects of Na_2_S, GYY4137, and AP39 were achieved by Ravani et al. [113], who additionally demonstrated the additive effects of the co-administration of donors with different mechanisms of action (Na_2_S/GYY4137 and Na_2_S/AP39) on the extent of I/R damage. Noteworthy, the regulation of NO bioavailability by H_2_S may also result from S-sulfhydration. The treatment of H9C2 cardiomyocytes with 100 μM of Na_2_S for 30 min increases the post-translational modification of protein tyrosine kinase 2 (PYK2), a member of the family of nonreceptor PYKs, known to be involved in the regulation of endothelial nitric oxide synthase (eNOS) activity. H_2_S-triggered PYK2 modification limits its activity and hence promotes eNOS function and NO generation [114]. 

In the anthracycline-induced cardiotoxicity model, NaHS was demonstrated to decrease lipid peroxidation, mitochondrial damage, and iron accumulation [72]. Importantly, doxorubicin-induced ferroptosis was attenuated by H_2_S through the S-sulfhydration of mitochondrial membrane protein optic atrophy 3 (OPA3), required for mitochondrial dynamic regulation. This prevented the ubiquitin-proteasome-mediated degradation of OPA3 and, through interaction with the cysteine desulfurase NFS1, which potentiates ferroptosis, led to the alleviation of doxorubicin-induced cell death [72].

Another micropeptide protein sulfhydrated by H_2_S is phospholamban (PLN) (Figure 4). NaHS regulates this modulator of Ca^2+^ uptake, which affects the activity of Ca^2+^-ATPase (SERCA2a) and modulates cardiac contractility and relaxation [115]. SERCA2a is known to be SUMOylated, and this post-translational modification is crucial for the SERCA2a-mediated regulation of myocardial contraction through an active reuptake of cytoplasmic Ca^2+^. Peng et al. [116] found an increase in SERCA2a SUMOylation resulting from the S-sulfhydration of the 683 cysteine site in sentrin-specific protease 1 (SENP1). This was accompanied by improved myocardial function in diabetic db/db mice and decreased cardiomyocyte apoptosis [116] (Figure 4). 

The modification of this and other specific proteins, discussed in the following paragraphs, contributes to H_2_S-triggered cardiovascular protection.

### 5.3. H_2_S Downregulates Oxidative Stress

The indication of the antioxidant properties of H_2_S may come from studies performed on CSE knock-out animals, characterized by accelerated oxidative stress and dysfunctional mitochondria [117]. On the other hand, CSE overexpression or stimulation with H_2_S donors attenuated oxidative-triggered cardiac muscle damage [62,118]. The main mechanisms involved in the regulation of oxidative status by H_2_S are related to direct ROS [119] and reactive nitrogen species (RNS) [120], quenching and increasing the level of antioxidant factors, including reduced glutathione (GSH). GSH, a tripeptide, γ-L-glutamyl-L-cysteinylglycine, is synthesized in consecutive reactions catalyzed by two enzymes, glutamate cysteine ligase (GCL) and glutathione synthetase (GS). H_2_S improves the transport of the sulfur amino acid precursor, cysteine, to increase GSH production. Moreover, elevated intracellular GSH levels result from H_2_S-induced upregulation of the expression of the GCL subunits, a catalytic subunit (GCLC), and a modifier subunit (GCLM) [121]. By modulating the concentration of GSH, H_2_S can efficiently ameliorate ROS-triggered lipid peroxidation, protein oxidation, and DNA damage. Indeed, in the isoproterenol-induced myocardial injury model, NaHS treatment decreased lipid peroxidation by scavenging superoxide anions (O_2_^−^) and H_2_O_2_ [118]. 

The molecular mechanisms of H_2_S as an anti-oxidant compound may also include sulfhydration [122] (Figure 4). An example of such modification is the activation of the nuclear factor erythroid 2-related factor 2 (NRF2; encoded by the *Nfe2l2* gene), which regulates the expression of numerous antioxidant genes [123,124]. H_2_S sulfhydrates Kelch-like ECH-associated protein 1 (KEAP1), a negative regulator of NRF2, resulting in a conformational change in KEAP1. This triggers a cascade of events leading to the accumulation of NRF2 in the nucleus and its binding to the antioxidant response element (ARE), as well as an induction of the expression of NRF2-regulated downstream genes [125,126,127]. Recent results also indicate that H_2_S (in the form of a NaHS donor) accelerated the S-sulfhydration of E3 ubiquitin-protein ligase synoviolin (Syvn1) at Cys115, which facilitates NRF2 nuclear translocation [128].

A plethora of genes are known to be NRF2-dependent, including heme oxygenase-1 (HO-1), thioredoxin (TRX), glutathione S-transferase (GST), glutathione peroxidase (GPx), thioredoxin reductase (TRXR), and catalase, and their activation leads to the mitigation of oxidative stress [129]. The regulation of these antioxidant proteins by H_2_S has been demonstrated as a cytoprotective mechanism ameliorating various pathological conditions, including cardiovascular disorders. In rats with volume overload-induced heart failure [130] and mice with coxsackievirus B3-induced myocarditis [131], the increased expression of HO-1 by H_2_S has been evident. Treatment with Na_2_S during acute MI led to the induction of NRF2 and its downstream targets, HO-1 and TRX, in ischemic cardiac tissue, which resulted in a reduction in infarct size, a marker for the level of cardiac injury, troponin I, and oxidative stress [60]. Na_2_S was also able to preserve cardiac structure and functions in wild-type but not NRF2 knock-out mice after ischemia-induced heart failure, suggesting an NRF2-dependent mechanism of action [132]. Furthermore, the involvement of NRF2 in NaHS activity was also underlined in a rat model of doxorubicin-induced cardiomyopathy. A NaHS solution administered at a dose of 15 μmol/kg/d for 12 weeks reduced ROS generation and malondialdehyde (MDA) levels; activated the NRF2 pathway; upregulated the expression of antioxidant proteins, NAD(P)H:quinone oxidoreductase 1 (NQO1) and GCLM; and increased superoxide dismutase (SOD) and GPx activities [133].

The activation of the NRF2-dependent pathway and its downstream regulated antioxidant enzymes by H_2_S was also demonstrated in cardiomyocytes in vitro. Tsai et al. [79] reported the upregulation of the NRF2 pathway in hyperglycemic H9C2 cells treated with DATS, the sulfur-containing compound found in garlic. The administration of DATS reduced the high-glucose-increased caspase-3 activity and ROS generation, but only when NRF2 was present [79]. Also, in isolated rat cardiomyocytes, NaHS decreased ROS generation, increased SOD activity (both mitochondrial Mn-SOD and cytoplasmic CuZn-SOD), and inhibited the activity of mitochondrial complex IV [134]. Additionally, SG-1002, the long-acting H_2_S prodrug, exerted cardioprotective activities on murine HL-1 cardiac muscle cells through an anti-oxidant mechanism [74]. In stressed cardiomyocytes (serum-starved or H_2_O_2_-treated), SG-1002 significantly increased *Cbs* expression, upregulated H_2_S levels, enhanced cell survival, and reduced cytotoxicity. Importantly, the antioxidant effects of SG-1002, through the upregulation of SOD and catalase and lowering the levels of advanced oxidative protein products (AOPPs) and ROS generation, have also been noted [74].

Not only is the KEAP1 protein sulfhydrated, leading to NRF2 activation to suppress ROS production and oxidative damage, but another example might be the p66Shc adaptor protein that plays a crucial role in mitochondrial redox signaling (Figure 4). When phosphorylated at Ser36, p66Shc is translocated to mitochondria, where it facilitates ROS production [135,136]. H_2_S may prevent this modification via sulfhydration at Cys59, and in consequence, the pro-oxidant effect of p66Shc is abolished. Both NaHS treatment and an overexpression of CBS resulted in the blocking of p66Shc Ser36 phosphorylation and the inhibition of ROS production. Furthermore, in cells with an introduced C59S mutation (cysteine 59 was replaced with serine), the beneficial effects of NaHS were abolished [137]. 

### 5.4. H_2_S Has Anti-Inflammatory Functions

In various pathological situations, the infiltration of inflammatory cells followed by fibrotic tissue accumulation occurs. H_2_S may exert powerful, noteworthy anti-inflammatory effects. Although various molecular pathways may be involved, the main mechanism is based on the inhibition of the activation and nuclear translocation of NF-κB, a central mediator of inflammation [138,139]. The mechanism behind this effect relies on the modification of the sulfhydryl group of a reactive cysteine 38 in the p65 subunit of NF-κB into a hydropersulfide group, which results in an anti-inflammatory effect [140] (Figure 4). As this factor controls the transcription of many pro-inflammatory genes, the effect of H_2_S on inflammation could be manifested via the changes in the expression of several factors. Indeed, in a porcine model of myocardial I/R injury (generated through mid-left anterior descending coronary artery occlusion for 60 min, followed by reperfusion for 120 min), the application of Na_2_S 10 min before and throughout reperfusion resulted in a decrease in tissue levels of TNF-α, IL-6, IL-8, and myeloperoxidase activity. These anti-inflammatory properties may contribute greatly to the beneficial effects of H_2_S supplementation on myocardial function, coronary microvascular reactivity, and infarct size [141]. 

An important anti-inflammatory mechanism of H_2_S has been proposed by Zhang et al. [142] and Wu et al. [143]. In these studies, a mouse model of heart failure induced by a permanent ligation of the left coronary artery was established, and NaHS administration reduced the recruitment of CD11b^+^Gr-1^+^ myeloid cells from the spleen and bone marrow to the myocardium [142,143]. Other possible mechanisms may also involve the regulation of IL-10/JAK/STAT3-dependent signaling [144]. Finally, the involvement of specific microRNAs, for example, the upregulation of cytoprotective miR-21, was underlined. In the work performed by Toldo et al. [145], Na_2_S significantly reduced infarct size in wild-type but not in miR-21 knock-out mice. Of note, Na_2_S-induced miR-21 expression resulted in a reduced inflammatory cell infiltration into the myocardium and decreased the formation of inflammasomes, a macromolecular structure responsible for the activation of inflammatory responses, formed by apoptosis speck-like proteins containing a caspase-recruitment domain (ASC), cryopyrin (or NLRP3), and caspase-1.

### 5.5. H_2_S Is Anti-Fibrotic

It is evident that the low H_2_S production resulting from a deficiency in CSE results in the development of fibrosis, and the severity of cardiac fibrosis is substantially correlated with a drop in H_2_S concentration in heart tissues [146,147,148,149]. On the other hand, H_2_S donor administration attenuates fibrosis progression [143,146,148,150,151,152,153]. The use of a liposomal formulation of S-propargyl-cysteine, resulting in a low release of the active gasotransmitter through the inhibition of the transforming growth factor-β1 (TGF-β1)/SMAD signaling pathway, exerted cardioprotective and anti-fibrotic effects [151]. Similarly, when another H_2_S donor, GYY4137, was used in spontaneously hypertensive rats (SHRs), a lower extent of MI was detected [154]. In another study utilizing the rat MI model, the delivery of NaHS resulted not only in a reduced area of infarction and a lower level of pro-fibrotic markers, including type I collagen, type III collagen, and matrix metalloproteinase-9 (MMP-9), but also improved heart functions [148]. Similarly, Wu et al. [143] found the protective effects of NaHS on myocardial fibrosis and cardiac remodeling following MI in mice. Comparable effects have been obtained with the use of NaHS in a rat model of angiotensin II-induced hypertensive heart disease [146]. In a volume overload-induced murine model of cardiac fibrosis, the expression of CSE protein and myocardial H_2_S production were significantly reduced, while NaHS treatment regulated the activity of MMPs and tissue inhibitors of metalloproteinases (TIMPs), playing a pivotal role in extracellular matrix remodeling, and attenuated tissue fibrosis [152]. The effect of NaHS supplementation was also tested in aged rats [155]. When 23-month-old rats with developed heart fibrosis were treated with NaHS, the fibrotic area (assessed via Masson’s trichrome staining and an evaluation of the hydroxyproline content) and the mRNA level of collagen I and α-SMA were reduced. Of note, an analogous result was found after 12-week-long moderate-intensity exercise training. It was suggested that the beneficial cardioprotective effects of exercise could be related to the restoration of the bioavailability of H_2_S in the heart, since the upregulation of CSE and MPST expression was evident. Moreover, after exercise, an increase in H_2_S levels in the plasma and heart by 39.8% and 90.9%, respectively, was detected [155]. 

During DMD progression, increased levels of fibrotic markers, like periostin (POSTN) and/or osteopontin (OPN), have been noted. A high abundance of POSTN, the non-structural matricellular protein that functions upstream and downstream of TGF-β, was found in the dystrophic diaphragm [156,157] as well as in the heart [158]. Additionally, it was strongly induced in other models of cardiac impairment, such as myocardial infarction and left ventricular pressure overload [159,160]. Interestingly, H_2_S has been demonstrated to reduce POSTN expression in vitro in mouse glomerular endothelial cells exposed to high glucose concentrations [161]; however, no data on the effect of H_2_S supplementation in the heart are available. In contrast, we have demonstrated that NaHS treatment leads to a reduction in the OPN level in the plasma and muscles (gastrocnemius, tibialis anterior, and diaphragm) of *mdx* mice [162]. The role of OPN in the progression of cardiovascular diseases and heart failure is undeniable, and it was demonstrated in studies utilizing OPN-null mice and experiments with its depletion by neutralizing antibodies or via targeted mutagenesis (reviewed in [163,164]). It may also significantly influence DMD-related cardiomyopathy [165]. More studies are warranted to discover the potential protective role of H_2_S in the attenuation of OPN-triggered cardiac inflammation and fibrosis.

These and other results clearly demonstrate that exogenous H_2_S may prevent cardiac remodeling by suppressing extracellular matrix formation and the production of fibrotic cytokines.

### 5.6. H_2_S Promotes Angiogenesis

H_2_S is also known to exert proangiogenic activities. It serves as an endogenous stimulator of angiogenesis through the regulation of various pathways, including the activation of K_ATP_ channels [85,166], the regulation of vascular endothelial growth factor (VEGF) signaling through AKT activation [167], or the hypoxia-inducible factor-1α (HIF-1α) pathways [168,169]. The involvement of MMPs in VEGF regulation was also underlined [170]. Additionally, the receptors involved in mediating VEGF activity, namely fms-like tyrosine kinase 1 (Flk1, VEGFR-1) and tyrosine kinase receptor (Flt1, VEGFR-2), were upregulated by NaHS in mice after MI [171]. Tao et al. identified a specific Cys1045-Cys1024 disulfide bond acting as an inhibitory motif prone to a nucleophilic attack of H_2_S. Targeting this motif upregulates VEGFR-2 functions and has a proangiogenic effect [172]. Not only proangiogenic factors are stimulated by H_2_S. The increase in angiogenesis might result from the inhibition of antiangiogenic molecules, for example, endostatin, angiostatin, and parstatin found in MI-induced heart failure after NaHS treatment [171]. A similar downregulation of endostatin and angiostatin, as well as MMP-9/TIMP3 signaling, by NaHS was cardioprotective during pressure-overload heart damage [170]. Lower angiostatin levels with concomitant upregulation in VEGF expression, as well as increased phosphorylation of eNOS and bioavailability of NO, were observed after DATS delivery 24 h after transverse aortic constriction [173]. An upregulation of eNOS resulting in protection against pressure overload-induced heart failure was demonstrated by Kondo et al. [174]. In this study, both CSE knock-out mice and cardiac-specific CSE transgenic animals were used to underline the role of H_2_S in the prevention of cardiac functions with, a special emphasis on its role in myocardial vascularization.

H_2_S may stimulate endothelial cell migration and proliferation and enhance morphogenesis in cultured endothelial cells, as demonstrated by Cai et al. [175]. Not only AKT-dependent tube formation by endothelial cells in vitro was increased when the cells were seeded on Matrigel, but also, in vivo, in a Matrigel plug assay in mice, accelerated neovascularization after NaHS treatment was evident [175]. In addition, the regulation of specific microRNAs, such as miR-192 [176] or miR-126-3p [177], may be responsible for the H_2_S-dependent promotion of angiogenesis.

The angiogenic properties of H_2_S may be also related to its S-sulfhydration activity. Specific protein 1 (SP-1), containing 11 cysteine residues, which may be hypothetically targeted by H_2_S, is a transcription factor with well-defined functions in the cardiovascular system. In human umbilical vein endothelial cells (HUVECs), the S-sulfhydration of SP-1 at Cys68 and Cys755 was shown to be crucial for maintaining the angiogenic activity and proper functions of endothelial cells [178]. The silencing of CBS or pharmacological inhibition of H_2_S production reduced S-sulfhydration, leading to an increased proteasomal degradation of SP-1, resulting in the downregulation of its binding to VEGFR-2 and the inhibition of endothelial cell proliferation and migration. On the other hand, NaHS supplementation increased SP-1 sulfhydration 2-fold and rescued the endothelial properties of HUVECs [178]. Another H_2_S donor, GYY4137, induced the S-sulfhydration of SP-1, not only in vitro in neonatal rat cardiomyocytes, but also in vivo in the myocardium of SHR animals [179]. In this case, the modification of Cys664 abolished the Krüppel-like factor 5 (KLF5) promoter binding to SP-1 and inhibited cardiac hypertrophy (because when SP-1 is coupled with KLF5 promoter, it has a pro-hypertrophic effect in the heart) (Figure 4).

### 5.7. H_2_S Protects against Apoptosis

The anti-apoptotic activities of H_2_S may be of great importance for cardioprotection and may involve the activation of the reperfusion injury salvage kinase (RISK) pathway, composed of two pro-survival kinases: PI3K and extracellular signal-regulated kinases (ERKs; ERK1/2) [180]. Another mechanism is based on the S-sulfhydration of NF-κB, which may not only inhibit inflammation, as discussed above. The conversion of an -SH group of Cys58 to an -SSH in the p65 subunit enhances its binding to the ribosomal protein S3 (RPS3) co-activator to activate the promoter of anti-apoptotic genes, and this contributes to the anti-apoptotic effect of H_2_S [181] (Figure 4).

In rat cardiomyocytes treated with NaHS, the phosphorylation of ERK1/2 and AKT was induced, while the blockade of these signaling pathways abolished the cardioprotective effects of H_2_S against ischemia [182]. The stimulation of cardiomyocytes subjected to 6 h of hypoxia and 12 h of reoxygenation with Na_2_S at the time of reoxygenation reduced apoptosis, shown through a decreased caspase-3 activity [183]. Liu et al. [71] also found that the beneficial effect of NaHS is mediated through the PI3K/AKT/FoxO3a pathway. In this study, exogenous H_2_S mitigated inflammation, cytotoxicity, oxidative stress, and apoptosis triggered by doxorubicin. Another in vitro cardiotoxicity model, hyperglycemia-induced cardiac dysfunction representing heart complications in diabetic patients, was applied by Huang et al. [73]. When H9C2 cardiac cells were cultured in high-glucose conditions (33 mM; 24 h), pro-inflammatory and proapoptotic pathways were induced. In contrast, pre-treatment with NaHS attenuated p38 MAPK/NF-κB-mediated inflammation and decreased caspase-3 expression. Furthermore, high-glucose-induced ROS generation and the dissipation of the mitochondrial membrane potential were attenuated. Similarly, in hyperglycemic H9C2 cells treated with garlic-derived DATS, the inhibition of PI3K/AKT signaling by LY294002 (PI3K inhibitor) or PI3K-specific siRNA resulted in the hampering of H_2_S effects [79].

Among various signaling pathways, the phosphorylation of glycogen synthase kinase-3beta (GSK-3β) on serine 9 was shown to exert cardioprotective activities. Both in vitro and in a rat model of ischemia/reperfusion, by inducing GSK-3β phosphorylation and reducing proapoptotic Bax translocation and caspase-3 activation, NaHS resulted in the inhibition of mitochondrial MPTP opening and cardiomyocyte apoptosis [184]. The regulation of the GSK-3β pathway was also shown to affect the autophagy process. In neonatal rat cardiomyocytes subjected to hypoxia/reoxygenation, NaHS enhanced cell viability and reduced autophagy. When PI3K activity was blocked with LY294002 or the activity of serum- and glucocorticoid-responsive kinase-1 (SGK1) was decreased by specific siRNA, the anti-autophagy effect of NaHS was blunted, suggesting the PI3K/SGK1/GSK3β signaling pathway’s contribution to the cardioprotective effect of H_2_S [185]. The modification of GSK-3β is not only restricted to the NaHS donor. Karwi et al. [186] tested GYY4137 for the treatment of acute MI. When given specifically at reperfusion, GYY4137 activated PI3K/AKT signaling and increased p-Ser9-GSK-3β, with cardioprotective effects. 

Also in vivo, in a rat model of doxorubicin-induced cardiomyopathy, a NaHS solution (15 μmol/kg/d) given for 12 weeks attenuated apoptosis in the myocardium by activating the PI3K/AKT pathway [133]. Zhang et al. [142] underlined the regulation of the apoptotic Bax/Bcl-2 proteins by NaHS in a mouse model of MI. Additionally, activated caspase-3 levels and the percentage of TUNEL-positive (apoptotic) cardiomyocytes in the infarcted heart were lower in animals treated with NaHS than in the mice with MI after saline delivery. Similarly, the increase in the expression of Bcl-2 and Bcl-xL, with decreased Bad levels, were evident after Na_2_S administration to mice subjected to I/R injury [60]. In this work, the phosphorylation of ERK1/2 and transducers and activators of transcription-3 (STAT3) by H_2_S was also evident. 

## 6. H_2_S as a (Cardio)Protective Factor in DMD

Until recently, the effect of H_2_S in DMD has not been studied. However, considering its biological properties and the possibility of modulating the myriad of processes contributing to DMD progression, it is not surprising that attention has been paid to this endogenously generated biological gasotransmitter.

Several studies have underlined alterations in the expression of H_2_S-generating enzymes and deficits in H_2_S production in DMD models. Panza et al. [187] found a lower expression of CSE and MPST in human primary myoblasts isolated from healthy and DMD donors. Moreover, a similar regulation was found in the quadriceps and gastrocnemius of dystrophic *mdx* mice in comparison to control animals. We also demonstrated the decreased mRNA and protein expression of H_2_S-generating enzymes in various muscles of 12-week-old *mdx* mice [162]. Of note, when we performed a proteomic analysis of the diaphragm of younger, 5–6-week-old animals, a lower abundance of CTH was found. This effect was confirmed through real-time and Western blot analyses, and additionally, we detected the decreased mRNA of Cbs and Mpst, as well as a reduced MPST protein level [156]. In line with these results, both dystrophic Caenorhabditis elegans (dys-1(eg33) mutant) [188,189] and mouse models [162,187,190] of DMD have demonstrated the usefulness of H_2_S donors in attenuating the severity of the disease. Ellwood et al. [188] underlined the H_2_S deficit as a key contributor to DMD progression in dystrophic worms. On the other hand, they suggested that delivering H_2_S donors (sodium GYY4137-NaGYY and AP39) has protective effects on the *C. elegens* healthy life span, including improved locomotion and strength and mitochondrial structure in muscles. The effects were comparable to the gold-standard treatment with the glucocorticoid prednisone [188]. Accordingly, when dys-1(eg33) animals were kept on nematode growth medium plates supplemented with sulfur-containing amino acids (L-methionine, L-homocysteine, L-cysteine, L-glutathione, and L-taurine), an improvement in movement through better muscle calcium handling and excitation–contraction coupling was observed [189]. Although the undeniable effect of different methods of H_2_S delivery on skeletal muscle has been proven, its role in the cardiac system remains unresolved in vivo as nematodes lack a circular system. Interestingly, the authors investigated the effect of NaGYY delivery on the contractile activity of the pharynx, a muscular pump involved in feeding, which has been suggested to share functional and molecular similarities with the heart in other species. Electropharyngeogram recordings demonstrated that the frequency of pumping, the pump duration, or the interpump interval of the pharynx in dystrophic worms after H_2_S supplementation was improved, but did not reach the level observed in WT animals [188]. Nevertheless, it has to be underlined that suggested similarities between the nematode pharynx and vertebrate heart represent a convergent evolution rather than true homology, and experiments on vertebrates are needed to analyze the possible cardioprotective effects of H_2_S in DMD. Similarly, the administration of H_2_S donors to *mdx* mice has been proposed as a therapeutic strategy for the treatment of DMD [162,187,190]. In these studies, slightly different protocols for NaHS delivery (dose, duration of supplementation, age of animals) resulted in the attenuation (to various degrees) of the typical DMD pathological insults, like the inflammatory and fibrotic status. The exogenous donors also regulated markers of oxidative stress and autophagy. In these works, the effectiveness of the H_2_S donor is analyzed in skeletal muscles, including the gastrocnemius, diaphragm, tibialis anterior, and anterior femoral quadriceps [162,187,190], but the condition and physiology of cardiac muscle have not been tested.

**Figure 5:**
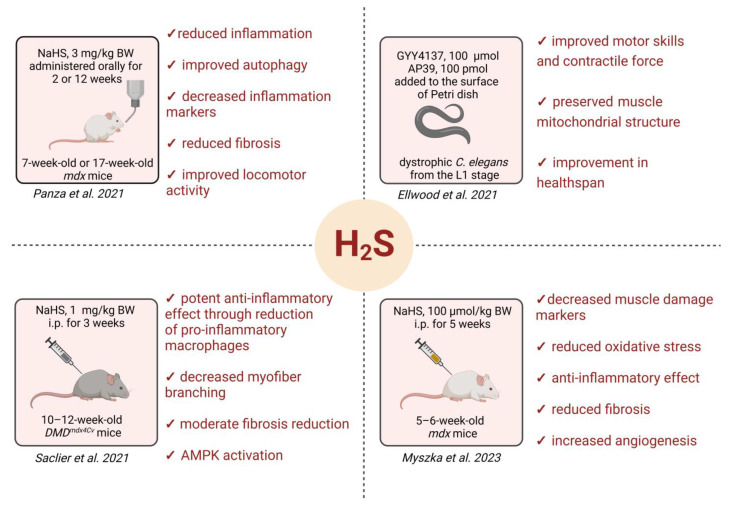
H_2_S in DMD—a summary of published research. Knowledge about the role of H_2_S in DMD is limited. Recent papers by Panza et al. [187], Saclier et al. [190], Ellwood et al. [188], and Myszka et al. [162] underline the potential therapeutic application of H_2_S donors to hamper disease progression. The main mechanisms that improve the dystrophic muscle phenotype identified in these studies are highlighted.

Overall, these results all suggest that overcoming the deficit of H_2_S in DMD models attenuates disease progression (summarized in Figure 5), but further studies are needed to investigate its possible role in the cardiac system. It is important to recall that, paradoxically, improvements in patient daily care and the use of ventilatory assistance may contribute to a proportionate rise in cardiac causes of death over those linked with respiratory failure. The paucity of studies examining the cardioprotective benefits of H_2_S donors in DMD, very probable to be true, might be disappointing, but it may be only a matter of time until such data are released. One of the reasons may be that the onset and extent of cardiac malfunction in the most commonly used mouse DMD model, *mdx* mice, do not recapitulate human pathology as mentioned above.

## 7. Conclusions and Future Perspectives

**Figure 6:**
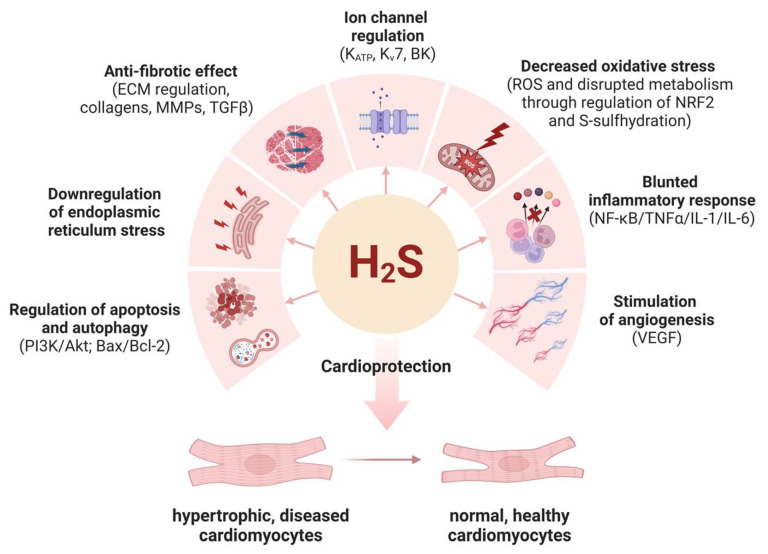
Cardioprotective effects of hydrogen sulfide. H_2_S acts as a pleiotropic factor, regulating the activity of ion channels, apoptosis/autophagy, and endoplasmic reticulum stress, exerting anti-fibrotic, anti-oxidative, and anti-inflammatory properties, together with proangiogenic functions. Examples of possible molecular mechanisms/main factors regulated by H_2_S are indicated in brackets and described in the text.

Each year, around 20,000 children worldwide are estimated to be diagnosed with DMD (https://www.mda.org/sites/default/files/2019/03/Duchenne_Muscular_Dystrophy_Fact_Sheet.pdf, accessed on 19 December 2023). Although progress in understanding the both mechanical and nonmechanical roles of dystrophin has been made, and recently, a gene therapy-derived drug has been accepted by the FDA, the current studies are focused on the evaluation of new targets and possible disease modulators. DMD patients die because of heart dysfunction. H_2_S, produced in the heart tissue through the activity of CSE, maintains cardiac homeostasis through anti-apoptotic, anti-inflammatory, and antioxidant properties, summarized in Figure 6. The ongoing explosive research on new H_2_S donor formulations may hopefully shortly lead to the discovery of potent drugs used to treat cardiovascular, pulmonary, and neurodegenerative disorders, as well as myopathies.

## Figures and Tables

**Table 1:** Cardioprotective drugs used for the treatment of DMD patients.

Type of Drugs	Abbreviation	Exemplary Drugs	Mechanism of Action	Recommendations for Drug Use	References
angiotensin-converting enzyme inhibitors	ACEis	perindopril, enalapril, captopril	inhibition of Ang II formation and metabolism	ACEis should be used even in asymptomatic DMD boys with normal LV systolic function by the age of 10 years	[16,17,18,21]
angiotensin receptor blockers	ARBs	losartan	competitive inhibition of Ang II binding to the angiotensin 1 receptor	ARBs should be used even in asymptomatic DMD boys with normal LV systolic function by the age of 10 years	[19,20,21]
mineralocorticoid receptor antagonists (aldosterone antagonists)	MRAs	eplerenone, spironolactone	blocking the endogenous MR, aldosterone, at its receptors	early MRA treatment can increase the chance of improving the cardiac condition	[22,23,24]
beta-adrenergic receptor	β-AR	bisoprolol, metoprolol, carvedilol	nonselective or selective inhibition of β-adrenergic receptor	second-line therapy in patients with tachycardia and/or no effect of ACEis	[25,26,27]

Abbreviations: Ang II, angiotensin II; DMD, Duchenne muscular dystrophy; LV, left ventricular; MR, mineralocorticoid.

**Table 2:** The selected cardioprotective effects of hydrogen sulfide in in vitro models.

Cardiac Disease	Cellular Model	Type and Concentration of H_2_S Donor	Time of Stimulation with H_2_S Donor	Additional Information about Cell Stimulation	Major Molecular Mechanism of H_2_S-Mediated Cardioprotection	References
DCM	Rat H9C2 cells; HG-induced cardiotoxicity model (33 mM glucose; 48 h)	NaHS (50 µM), SPRC (5–25 µM)	4 h	Cells were pre-treated with H_2_S donors for 4 h before culturing in HG medium	Activation of AKT/NRF2	[78]
DCM	Rat H9C2 cells; HG-induced cardiotoxicity model (33 mM glucose; 36 h)	DATS (1–10 µM)	12–48 h	Cells were treated with HG and DATS (1, 5, or 10 μM) for 36 h or DATS (10 μM) for 12–48 h	Activation of PI3K/AKT/NRF2	[79]
DCM	Rat H9C2 cells; HG-induced cardiotoxicity model (33 mM glucose; 24 h)	NaHS (400 µM)	30 min	Cells were pre-treated with 400 μM of NaHS for 30 min before culturing in HG medium	Suppression of TLR4/NF-κB pathway: alleviation of HG-induced activation of NLRP3 inflammasome, TLR4, and NF-κB	[70]
DCM	Rat H9C2 cells; HG-induced cardiotoxicity model (33 mM glucose; 24 h)	NaHS (400 µM)	30 min	Cells were pre-treated with 400 µM of NaHS for 30 min before culturing in HG medium	Inhibition of the p38MAPK/NF-κB, COX-2 and iNOS signaling pathways	[73]
DCM	Human AC16 cells; cardiac lipotoxicity model (500 µM PA; 24 h)	NaHS (100 µM)	24 h	NaHS treatment was repeated every 6 h during the entire treatment period of 24 h	Inhibition of ER stress: downregulation of stress marker proteins including GRP78, CHOP, and caspase-12	[76]
I/R injury	Rat H9C2 cells; hypoxia/reoxygenation model (H/R model: 0.1% O_2_ + 5% CO_2_ in 1% FBS serum-starvation medium for 4 h. After hypoxia, the cells were re-oxygenated in 95% O_2_ + 5% CO_2_)	NaHS (200 µM)	Not specified directly	Initially, H_2_S in different concentrations from 50 to 200 μM were tested, and then 200 μM was used for subsequent experiments	Inhibition of ER stress: downregulation of stress marker proteins including GRP78, CHOP, and eIF2α. The involvement of miR-133a in the H_2_S effect was demonstrated.	[80]
I/R injury	Old rat H9C2 cells (aging: 30 μM of H_2_O_2_; 2 h and subsequent culture for 3 days); hypoxia/reoxygenation model (H/R model: aged cardiac cells were exposed to a hypoxic culture medium for 3 h and reoxygenated for 6 h)	NaHS (100 µM)	6 h	NaHS was added for a 6 h reoxygenation phase	Inhibition of ER stress: decreased the expression of GRP78, CHOP, cleaved caspase-12, ATF4, ATF6, and XBP-1, and the phosphorylation of PERK, eIF2α, and IRE1α	[81]
HHcy-induced MI	Rat H9C2 cells; hyperhomocysteine-induced ER stress model (Hcy 0.1–2.5 mM; 6 h)	NaHS (100–1000 µM)	30 min	Cells were pre-treated for 30 min with NaHS and then supplemented with Hcy for 6 h	Inhibition of ER stress: decreased CHOP expression induced by Hcy	[82]
DOX-induced cardiotoxicity	Rat H9C2 cells (5 µM DOX; 24 h)	NaHS (400 µM)	30 min	Cells were treated with NaHS for 30 min before exposure to DOX	Inhibition of ER stress: blocking of DOX-induced overexpression of GRP78 and CHOP	[83]
DOX-induced cardiotoxicity	Rat H9C2 cells (5 µM DOX; 60 min)	NaHS (400 µM)	30 min	H9c2 cells were pre-treated with NaHS for 30 min before DOX treatment	Decreased expression level of phospho-p38 MAPK	[84]
DOX-induced cardiotoxicity	Rat H9C2 cells (5 µM DOX; 24 h)	NaHS (100 µM)	30 min	Cells were pre-treated with NaHS for 30 min, followed by exposure to DOX for 24 h	Activation of PI3K/AKT/FoxO3a pathways	[71]
DOX-induced cardiotoxicity	Rat H9C2 cells (1 μM DOX; 24 h)	NaHS (30 μM)	30 min	Cells were pre-treated NaHS for 30 min, then the supernatant was substituted with medium containing DOX	Increased S-sulfhydration and downregulation of OPA3 ubiquitination	[72]
HF/cardiac hypertrophy	Murine HL-1 cells (starvation in 1% FBS-containing media; oxidative stress induction: 500 μM H_2_O_2_, 1 h)	SG-1002 (10 μM)	1 h	Serum-starved cells were treated for 1 h with SG-1002, H_2_O_2_, or in combination	Inhibition of oxidative stress: induction of antioxidant proteins, catalase and SOD1	[74]

Abbreviations: ATF4, activating transcription factor 4; CHOP, C/EBP homologous protein; DATS, diallyl trisulfide; DCM, diabetic cardiomyopathy; DOX, doxorubicin; eIF2α, eukaryotic translation initiation factor 2α; ER, endoplasmic reticulum; GRP78, glucose-regulated protein 78; Hcy, homocysteine; HHcy, hyperhomocysteinemia; HG, high glucose; HF, heart failure; H/R, hypoxia/reoxygenation; H_2_S, hydrogen sulfide; IRE1α, inositol-requiring enzyme 1α; I/R injury, ischemia/reperfusion injury; MI, myocardial infarction; miR-133a, microRNA-133a; Na_2_S, sodium disulfide; NaHS, sodium hydrosulfide; NRF2, nuclear factor E2-related factor 2; OPA3, optic atrophy 3 outer mitochondrial membrane lipid metabolism regulator; PA, palmitic acid; PERK, pancreatic endoplasmic reticulum kinase; PI3K, phosphatidylinositol 3-kinase; SOD1, superoxide dismutase; SPRC, S-propargyl-cysteine; XBP-1, X-box-binding protein 1.

## Data Availability

Not applicable.
